# Understanding the willingness of healthcare workers to treat viral infected patients in Saudi Arabia: evidence from post-COVID-19 pandemic

**DOI:** 10.3389/fsoc.2025.1461479

**Published:** 2025-03-04

**Authors:** Abdulhadi Sharhan Alotaibi

**Affiliations:** Department of Sociology and Social Work, Imam Mohammad Ibn Saud Islamic University (IMSIU), Riyadh, Saudi Arabia

**Keywords:** healthcare workers, willingness, treat, COVID-19, patients

## Abstract

During the recent COVID-19 pandemic, healthcare workers played an essential role in saving millions of lives and stopping the spread of the virus worldwide. This study investigates the impact of perceived behavioral control, attitudes, subjective norms, and emotion-focused coping on willingness to treat viral-infected patients in Saudi Arabia. However, the theory of planned behavior was extended by including emotion-focused coping. Data were collected from 283 male and female healthcare workers from public, private, and semi-government hospitals. “Structural Equation Modeling” (SEM) was applied to test the hypothetical relationship using SmartPLS software. Overall, the findings indicate that healthcare workers perceived behavioral control, subjective norms, and emotion-focused coping significantly impact healthcare workers’ willingness to treat viral-infected patients. In contrast, attitudes showed a negative effect. In addition, emotion-focused coping mediates the relationship between perceived behavioral control, subjective norms, and willingness to treat viral-infected patients; emotion-focused coping does not mediate the relationship between attitudes and willingness to treat viral-infected patients. Overall, findings suggested that healthcare workers showed positive perceived behavioral control, subjective norms, and emotion-focused coping toward viral-infected patients. On the other hand, due to the novelty of the viral-infected viruses, attitudes of healthcare workers toward willingness to treat viral-infected patients shows that healthcare workers feel stressed and scared to treat viral-infected patients.

## 1 Introduction

The outbreak of the novel coronavirus (COVID-19) has posed unprecedented challenges to healthcare systems worldwide, necessitating swift and adaptable responses from healthcare professionals across various disciplines (Spoorthy et al., 2020; Shaikh et al., 2022). Among these professionals, healthcare workers have provided psychosocial support, advocacy, and assistance to patients and their families during these times of crisis (Ness et al., 2021; Snoubar and Zengin, 2022). Their willingness and readiness to engage directly in the care of COVID-19 patients are crucial for ensuring comprehensive and effective healthcare delivery (McCready et al., 2023). However, the novelty of the virus has created a challenging environment, with some healthcare professionals hesitant to treat infected patients due to fear and uncertainty. This hesitance contrasts with their responsibility to treat and minimize the spread of the virus (Shaikh et al., 2022; Matta et al., 2023).

However, in Saudi Arabia, the healthcare landscape has rapidly evolved to manage the challenges posed by COVID-19 (Shaikh et al., 2022), which aims to save lives (Tripathi et al., 2020) and normalize the social and economic activities (Khan et al., 2021). As the pandemic continues to strain healthcare resources and infrastructure globally, understanding the perspectives and willingness of healthcare social workers in Saudi Arabia to treat COVID-19 patients becomes paramount. Recently, Wu et al. (2020) highlighted that the willingness of healthcare social workers to treat viral-infected patients is influenced by multifaceted factors, including personal beliefs, training adequacy, perceived risks, institutional support, and the broader socio-cultural context within which they operate. Despite the critical role of healthcare social workers in providing psychosocial support and advocacy, their willingness to directly treat viral-infected patients in Saudi Arabia remains underexplored (Matta et al., 2023). Understanding the factors influencing their readiness and the barriers they face is essential for optimizing healthcare delivery during past pandemics. Thus, there is a pressing need to investigate the willingness of healthcare social workers in Saudi Arabia to treat viral-infected patients to enhance their preparedness and effectiveness in responding to public health emergencies. The present study aims to investigate and conclude the willingness of healthcare workers to treat viral-infected patients in Saudi Arabia by employing the extended theory of planned behavior.

Notably, the global healthcare community continues to learn from the ongoing COVID-19 pandemic. The insights gained from this study are instrumental in strategizing policies and practices that bolster the resilience and preparedness of healthcare social workers in Saudi Arabia and beyond. Enhancing the willingness and capacity of healthcare social workers to treat patients infected with viruses is crucial for strengthening the overall healthcare response and ensuring comprehensive care for all individuals affected by the pandemic. This research provides valuable insights into the pivotal role of healthcare workers during the recent COVID-19 crisis, highlighting opportunities to foster their engagement in patient care. It contributes to the broader discourse on healthcare resilience in Saudi Arabia. By addressing these issues, the study aims to inform evidence-based strategies that support and empower healthcare workers in their mission to provide compassionate and effective care amid the challenges posed by pandemics.

In addition, this study explores and acknowledges the barriers that healthcare workers face in Saudi Arabia while treating viral-infected patients. These barriers may include personal safety, insufficient training, lack of resources, and cultural or social factors. Addressing these challenges is essential for developing targeted interventions and policies to support healthcare workers and optimize their patient care roles. Therefore, findings from the present study contribute to educating policymakers, healthcare institutions, and professional bodies on how to motivate healthcare workers to treat infected patients. Overall, findings from the present study strengthen support systems, improve training protocols, and enhance the resilience of healthcare social workers in responding to future pandemics or public health emergencies like Monkeypox.

## 2 Literature review and hypotheses development

This section employs and extends the theory of planned behavior to synthesize research on perceived behavioral control, attitudes, subjective norms, and emotion-focused coping, exploring their impact on healthcare professionals’ willingness to treat viral-infected patients. After identifying key trends and gaps in the existing literature, the present study notably proposes to test the direct and indirect hypotheses. These hypotheses examine how these psychological constructs influence decision-making processes in medical settings.

### 2.1 Perceived behavioral control, emotion-focused coping, and willingness to treat viral infected patients

Perceived behavioral control is a fundamental dimension of the theory of planned behavior, effectively predicting individuals’ perceptions of their ability to perform specific activities (Shubayr et al., 2020). Factors influencing perceived behavioral control include the availability of resources such as personal protective equipment, the adequacy of training, and perceived support from healthcare institutions. Prior research highlights that preventive and awareness behavior is vital for crafting customized interventions and aiding healthcare policymakers in identifying pandemic-related issues that require broad attention (Shubayr et al., 2020; Aschwanden et al., 2021). Ngwewondo et al. (2020) investigated and concluded that the healthcare staff, through a COVID-19 awareness and prevention program, exhibit positive emotions toward COVID-19 patients, motivating them to provide care. The emotions of healthcare professionals, whether favorable or unfavorable, play a significant role in their willingness to treat COVID-19 patients. However, due to the novelty of the pandemic, limited existing studies explore the key factors that motivate medical professionals to treat viral-infected patients during a pandemic (Riaz et al., 2020; AlMazeedi et al., 2020). Kaye et al. (2021) examined and confirmed a significant positive relationship between treating COVID-19 patients and the perceived efficacy of the pandemic response. Similarly, Saqlain et al. (2020) identified the novelty of the virus as a source of stress related to perceived efficacy, with fear playing a significant role among healthcare professionals. Following the above arguments and criticism, limited studies explore the link between perceived behavioral control and emotion-focused coping. Thus, the present study proposed the following hypothesis.

*H1*: Perceived behavioral control significantly and positively impacts emotion-focused coping.

### 2.2 Attitudes, emotion-focused coping, and willingness to treat viral infected patients

Attitude is a key dimension of the theory of planned behavior, which is defined as an individual’s liking or dislike of a particular behavior (Conner and Sparks, 2005; Shoaib and Saleem, 2023). In contrast, it is easier to describe a healthcare professional’s positive or negative emotions in treating viral-infected patients if their behavior is defined. Saqlain et al. (2020) highlight that most medical professionals feared treating COVID-19 patients because of the pandemic’s novelty and risk of getting infected, demonstrating their unfavorable emotions toward affected people. Nonetheless, earlier research by Abdel Wahed et al. (2020) and Huynh et al. (2020) found a significant correlation between attitudes and emotional readiness to treat COVID-19 patients. Accordingly, Limbu et al. (2020) discussed that medical professionals show a significant positive attitude toward COVID-19 patients. At the beginning of the epidemic, most healthcare professionals found it difficult to be optimistic with COVID-19 patients. Furthermore, Olum et al. (2020) concluded that treating COVID-19 patients during the pandemic in Uganda is considerably aided by the upbeat attitude of healthcare professionals. Moreover, Nguyen et al. (2021) demonstrated that healthcare professionals’ readiness to treat COVID-19 patients was compromised by uncertainties about which drugs to administer to positive patients as initial treatment and how to administer them properly. As a result, limited research has been carried out in developing countries, mainly Saudi Arabia. Therefore, this study proposed the following hypothesis to investigate the healthcare workers’ attitudes toward emotion-focused coping to treat viral infected patients.

*H2*: Attitude toward the viral infections pandemic significantly and positively impacts emotion-focused coping.

### 2.3 Subjective norms emotion-focused coping and willingness to treat viral infected patients

Subjective norms are defined as the influence of peers, family, and friends on how medical professionals handle COVID-19 patients (Patwary et al., 2021). Healthcare professionals are emotionally vulnerable to the COVID-19 virus due to its rapid transmission from person to person (Minuye et al., 2021). Despite the risk of infection, most healthcare professionals were often compelled by their social circles or personal inclinations to avoid treating or associating with COVID-19 patients (Sin and Rochelle, 2022). Empirical research has shown that subjective norms can influence healthcare professionals’ willingness to treat COVID-19 patients by shaping their emotional responses to the pandemic. In this context, Godbersen et al. (2020) observed that social norms encourage healthcare personnel to adhere to preventative measures such as maintaining social distance, using face masks, and frequently washing and sanitizing hands. Additionally, Aschwanden et al. (2021) found that housekeepers in healthcare settings are required to rigorously follow all preventive guidelines when treating or visiting patients afflicted with COVID-19. A study in Ethiopia by Minuye et al. (2021) further revealed that most healthcare personnel’s subjective norms positively inspire them to treat COVID-19 patients, highlighting the crucial role of healthcare professionals in saving lives during the pandemic by effectively communicating the risks involved. Therefore, despite the considerable danger of infection, healthcare professionals must continue to treat every COVID-19 patient with vigilance (Nguyen et al., 2021). Further, there is a lack of empirical studies that examine the relationship between subjective norms and emotion-focused coping strategies. Thus, the present study proposed the following hypothesis.

*H3*: Subjective norms toward the viral infections pandemic significantly and positively impact emotion-focused coping.

### 2.4 Emotion-focused coping and willingness to treat viral infected patients

Emotion-focused coping involves managing emotions rather than changing the stressor (Nikolaev et al., 2023). This emotional approach is particularly relevant in healthcare settings, where healthcare professionals often face high-stress situations, such as treating patients infected with viruses (Sharma et al., 2020). Understanding how emotion-focused coping influences healthcare professionals’ willingness to treat these patients can guide interventions to support them effectively. Riaz et al. (2020) found that emotion-focused coping positively impacts healthcare professionals’ willingness to treat patients, indicating that those with higher levels of emotion-focused coping are more likely to treat viral-infected patients. Additionally, Nikolaev et al. (2023) highlighted that coping strategies such as seeking social support and positive reinterpretation help healthcare professionals manage stress and maintain a positive attitude toward challenging pandemic situations, potentially enhancing their willingness to engage in treatment. However, there is limited research on the relationship between emotion-focused coping and the willingness to treat viral-infected patients, especially in developing countries. According to Nikolaev et al. (2023), “emotion-focused coping—behaviors and thoughts to merely make them feel better (e.g., venting and denial)—which can then promote eudaimonic well-being such as a sense of personal growth and meaning” (p. 2123). Emotion-focused coping involves regulating individuals’ positive or negative feelings and emotional responses to the problem instead of addressing the issue. In the present study, emotion-focused coping defines the medical social workers’ emotional response toward treating COVID-19 patients. Therefore, the present study is among the first studies proposing to examine the direct link between emotion-focused coping and willingness to treat viral-infected patients in Saudi Arabia. Hence, the present study suggested the following hypothesis.

*H4*: Emotion-focused coping significantly and positively impacts willingness to treat viral-infected patients.

### 2.5 The mediating role of emotion-focused coping

After the direct hypotheses, the present study aims to test the mediating role of emotion-focused coping between perceived behavioral control, attitudes toward the pandemic, subjective norms, and healthcare professionals’ willingness to treat COVID-19 patients. This coping mechanism, which involves regulating emotional responses rather than altering the stressful situation (Riaz et al., 2020), is particularly relevant in the high-stress context of a pandemic (Saqlain et al., 2020). For instance, perceived behavioral control, reflecting individuals’ beliefs in their capabilities to perform tasks, directly influences their willingness to act (Shaikh et al., 2022). However, the actual engagement in treating patients may depend on how these individuals manage the emotional toll such responsibilities entail. Similarly, attitudes toward the pandemic can significantly affect emotional states, shaping behavior (Aschwanden et al., 2021). Negative attitudes might reduce willingness unless effectively managed through emotion-focused coping (Zhong et al., 2024). Likewise, subjective norms influence perceived social pressures and expectations, enhancing or diminishing motivation depending on emotional coping strategies (Shaikh et al., 2022). Thus, emotion-focused coping is a crucial bridge, converting intrinsic beliefs and external pressures into practical readiness to face challenging healthcare tasks. Therefore, this study proposed the following mediating hypotheses.

*H5*: Emotion-focused coping mediates the relationship between perceived behavioral control and willingness to treat COVID-19 patients.

*H6*: Emotion-focused coping mediates the relationship between attitudes toward the pandemic and willingness to treat COVID-19 patients.

*H7*: Emotion-focused coping mediates the relationship between subjective norms toward the pandemic and willingness to treat COVID-19 patients.

### 2.6 Underpinning theory

The theory of planned behavior is a prominent psychological framework used to understand and predict human behavior in various contexts, including healthcare settings. Developed by Icek Ajzen in the late 1980s (Ajzen, 1991). The theory of planned behavior posits that an individual’s behavioral intentions are influenced by three main dimensions, i.e., attitudes, subjective norms, and perceived behavioral control (Fishbein and Ajzen, 2010). Montano and Kasprzyk (2015) stated that the theory of planned behavior builds upon its predecessor, the theory of reasoned action, by incorporating the concept of perceived behavioral control. The theory of reasoned action suggests that attitudes toward behavior and subjective norms drive behavioral intentions. Ajzen extended this theory to include perceived behavioral control, which reflects the perceived ease or difficulty of performing the behavior (Fishbein and Ajzen, 2010).

Empirically, the theory of planned behavior has been widely applied in healthcare research to investigate various behaviors among healthcare professionals, including their willingness to treat patients under specific conditions (Ko et al., 2004; Patwary et al., 2021; Shaikh et al., 2022). In the context of infectious diseases, such as during the COVID-19 pandemic, the theory of planned behavior has been particularly relevant in understanding healthcare workers’ attitudes and intentions toward treating infected patients. Practically, in Saudi Arabia, the COVID-19 pandemic has highlighted the importance of understanding healthcare workers’ willingness to treat viral-infected patients (Shubayr et al., 2020). Post-pandemic, applying the theory of planned behavior can provide valuable insights into the factors influencing the intentions of healthcare social workers in this regard. Post-pandemic, empirical studies like (Alshehri et al., 2024) applied the theory of planned behavior in Saudi Arabia to investigate healthcare workers’ willingness to treat viral-infected patients. Research findings informed healthcare policies and interventions to enhance healthcare workers’ readiness and willingness to respond to future infectious disease outbreaks.

Overall, the theory of planned behavior provides a robust framework for exploring the complex dynamics influencing healthcare social workers’ willingness to treat viral-infected patients in Saudi Arabia post-COVID-19 pandemic. By examining attitudes, subjective norms, and perceived behavioral control, researchers can gain valuable insights to support healthcare workforce preparedness and enhance patient care strategies in infectious disease contexts.

## 3 Methods

A close-ended questionnaire was administered in public, private, and semi-government hospitals in Riyadh, the capital city of Saudi Arabia, from June 2022 to February 2024. The ultimate motivation for choosing Riyadh as a survey site is that several healthcare professionals were affected in Riyadh while treating COVID-19 patients during the pandemic (Aleanizy et al., 2021). Furthermore, the number of positive cases of COVID-19 was higher in Riyadh than in other cities in Saudi Arabia (Alharbi et al., 2021). However, before starting this survey, the selected hospitals and COVID-19 facilities centers were visited to identify the distinctive features of participants working in these hospitals. Then, the top management was reached, and permission was requested from the targeted respondents to conduct this survey. In addition, before starting the survey, participants were briefed about the study’s objectives and informed that they could choose not to participate or withdraw at any point. Furthermore, the researchers followed ethical standards for human research, maintaining the anonymity and confidentiality of participants throughout the study. Using a convenience sampling approach, data was gathered solely via an online survey distributed through multiple platforms such as WhatsApp and email. According to Irfan et al. (2021), the convenience sample approach is more valuable and accessible for surveying specific contexts, such as experimental behavioral research.

### 3.1 Demographic characteristics

To obtain the aim of the present study, 300 questionnaires were distributed among healthcare professionals, from which 283 were returned. Demographically, 74.5% of respondents were male, and 24% were female; the majority of the age group was 36–45 years old, 43.9%. Furthermore, the demographic information of the respondents is presented in Table 1. All the demographic information was calculated utilizing SPSS software.

**Table 1:** Demographic characteristics.

	Items	Frequency	Percentage
Gender	Male	211	74.55
	Female	72	24.55
Age	18–25	33	11.60
	26–35	51	18.02
	36–45	102	36.04
	45 and above	97	34.27
Educational background	MBBS	167	59.01
	PG Diploma	97	34.27
	Other	19	6.71
Working sector	Public	193	68.19
	Private	23	8.12
	Semi-public and private	67	23.67
Working experience	<1 year	88	31.09
	1–3	54	19.08
	4–7	48	16.96
	7–10	64	22.61
	More than 10 years	29	10.24

### 3.2 Measurements

A research model based on the five constructs (i.e., attitudes, subjective norms, perceived behavioral control, emotion-focused coping, and willingness to treat viral-infected patients) was developed for the current study. Thus, the current study’s research model was developed using an extended theory of planned behavior. Additionally, attitudes, subjective norms, perceived behavioral control, and willingness to treat viral-infected patients measuring items were adapted from Shaikh et al. (2022), while emotion-focused coping was derived from Nikolaev et al. (2023). In the present study, behavioral disengagement was measured, as suggested by Nikolaev et al. (2023). Furthermore, the present study applied a static measure to measure coping behavior. As a result, the paper also made certain modifications considering the present study’s setting.

**Figure 1:**
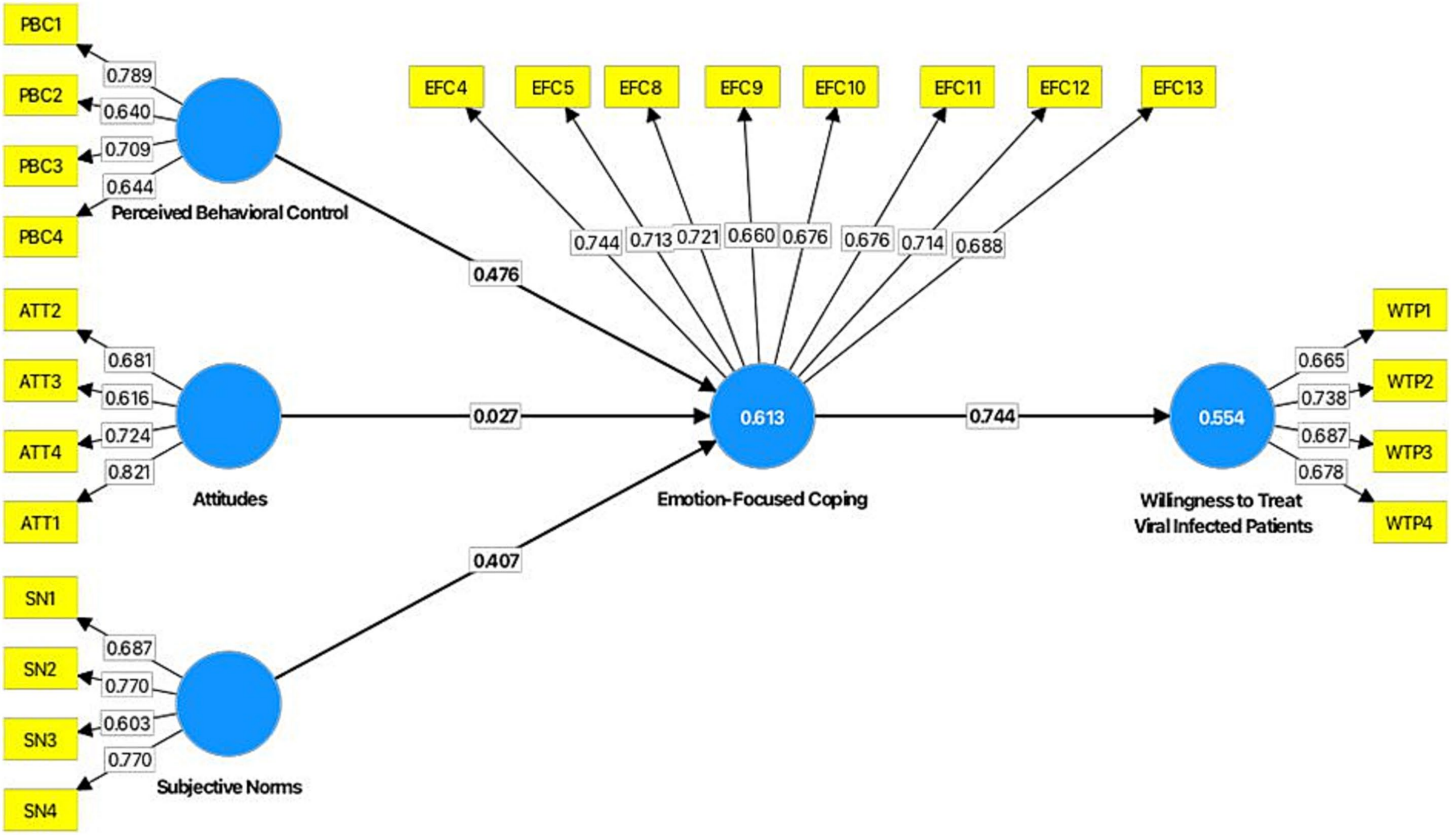
Structural model.

Additionally, the questions were measured using a five-point Likert scale (1 being strongly disagree and five being strongly agree) (Johns, 2010). According to Saleem et al. (2022), item factor loadings were considered >0.06. Hence, five items of emotion-focused coping loading values were lower than 0.06; thus, these items were deleted from consideration for the study’s hypothetical analysis (Saleem et al., 2023). Nonetheless, Cronbach’s Alpha ranged from 0.703 to 0.851, composite reliability (CR) ranged from 0.705 to 0.885, and finally, average variance extracted (AVE) from 0.501 to 0.510 were used to calculate the constructs’ reliability and validity tests. According to the statistical results, all constructs fit within the study’s parameters (Wong, 2013). Thus, Table 2 and Figure 1 present the model’s factor loading, reliability, and validity. Therefore, Table 3 and Table 4 indicate the present study model’s discriminant validity.

**Table 2:** Measurement items.

Constructs/items	Loading	*α*	CR	AVE
Perceived behavioral control		0.737	0.785	0.508
PBC1	0.789			
PBC2	0.640			
PBC3	0.709			
PBC4	0.644			
Attitudes		0.703	0.805	0.510
ATT1	0.681			
ATT2	0.616			
ATT3	0.724			
ATT4	0.821			
Subjective norms		0.670	0.802	0.770
SN1	0.687			
SN2	0.770			
SN3	0.603			
SN4	0.770			
Emotion-focused coping		0.851	0.885	0.501
EFC4	0.744			
EFC5	0.713			
EFC8	0.721			
EFC9	0.660			
EFC10	0.676			
EFC11	0.676			
EFC12	0.714			
EFC13	0.688			
Willingness to treat viral infected patients		0.739	0.786	0.509
WTP1	0.665			
WTP2	0.738			
WTP3	0.687			
WTP4	0.678			

**Table 3:** HTMT discriminate validity.

Constructs	1	2	3	4	5
Attitudes					
Emotion-focused coping	0.402				
Perceived behavioral control	0.447	0.816			
Subjective norms	0.611	0.880	0.852		
Willingness to treat_viral infected patients	0.643	0.891	0.893	0.803	

**Table 4:** Fronell-Larcker criterion discriminate validity.

Constructs	1	2	3	4	5
Attitudes	0.714				
Emotion-focused coping	0.344	0.700			
Perceived behavioral control	0.296	0.679	0.693		
Subjective norms	0.408	0.667	0.551	0.711	
Willingness to treat_viral infected patients	0.444	0.643	0.579	0.671	0.692

The results from Tables 3, 4 provide insight into the discriminant validity of the constructs under investigation, assessed through the Heterotrait-Monotrait Ratio (HTMT) and Fornell-Larcker criterion.

Table 3 highlights the HTMT values, a more rigorous approach to evaluate discriminant validity. The HTMT values for all construct pairs are below the conservative threshold of 0.85, except for emotion-focused coping with subjective norms (0.880) and willingness to treat viral-infected patients (0.891). These high values suggest potential issues with discriminant validity between these constructs, indicating they may overlap conceptually.

Table 4 presents the Fornell-Larcker criterion results, where the square root of the average variance extracted (AVE) for each construct (diagonal values) should exceed its correlations with other constructs. All constructs meet this criterion, implying an acceptable level of discriminant validity. For instance, attitudes (0.714) and subjective norms (0.711) exceed their correlations with other constructs, confirming that they capture distinct concepts.

## 4 Data analysis

The direct and indirect relationship between attitudes, subjective norms, perceived behavioral control, emotion-focused coping, and willingness to treat viral-infected patients was examined using structural equation modeling (SEM) using SmartPLS software. Crockett (2012) states that SEM assesses the study model and the structural coefficient path estimate. Researchers in the social and management sciences frequently use SEM methodologies to evaluate the validity and dependability of their research models (Saleem et al., 2022). Furthermore, the best methods, “covariance-based” (CB-SEM) and “partial least squares,” are often used to assess the study’s structural model. Consequently, PLS-SEM test procedures were used to examine the complex relationship between the constructs, provide route coefficient values, and support the theoretical techniques used in the study (Hair et al., 2017). Therefore, the direct and indirect relationships between attitudes, subjective norms, perceived behavioral control, emotion-focused coping, and willingness to treat viral-infected patients were ascertained using a bootstrapping approach with 5,000 subsamples and the t-statistic. In the structural model (Figure 1 and Table 4), path coefficients and coefficients of determination (R^2^) are finally displayed.

### 4.1 Hypotheses testing

To achieve the objectives of the present study, we developed five direct hypotheses and tested the estimated relationships using Partial Least Squares Structural Equation Modeling (PLS-SEM) through SmartPLS. Four of the five hypotheses were supported, and the overall statistical findings are detailed below.

The first hypothesis (H1) indicates that perceived behavioral control significantly predicts emotion-focused coping strategies among healthcare social workers where the (*t* = 4.96). Higher perceived control over their actions and decisions in treating viral-infected patients correlates positively with the tendency to adopt emotion-focused coping mechanisms.

The second hypothesis (H2) shows that the attitudes toward treating viral-infected patients do not significantly predict the use of emotion-focused coping strategies, and the statistical results are presented as (*t* = 0.048). This suggests that healthcare workers’ evaluations (attitudes) regarding patient treatment do not influence their coping strategies to manage emotional responses.

The third hypothesis (H3) statistically shows that the (*t* = 4.413) presents that the subjective norms, which reflect perceived social pressures and expectations, significantly predict emotion-focused coping strategies. Healthcare workers who perceive strong social norms regarding patient treatment are more likely to employ emotion-focused coping methods.

The fourth hypothesis (H4) illustrated that the (*t* = 11.688), which interpreted that the emotion-focused coping strategies significantly predict healthcare social workers’ willingness to treat viral-infected patients. Those who use more emotion-focused coping mechanisms are more inclined to express a willingness to engage in patient treatment despite the challenges posed by infectious diseases. Therefore, overall direct hypothetical results are presented in Table 5.

**Table 5:** Direct hypotheses.

Path	Original sample (O)	T statistics	*p-*values
Perceived behavioral control → Emotion-focused coping	0.442	4.960	0.000
Attitudes → Emotion-focused coping	0.048	0.736	0.462
Subjective norms → Emotion-focused coping	0.404	4.413	0.000
Emotion-focused coping → Willingness to treat viral infected patients	0.743	11.688	0.000

The present study also tested the mediating role of emotion-focused coping between perceived behavioral control, attitudes, and willingness to treat viral-infected patients. Therefore, we developed three mediating hypotheses, which are discussed below.

The fifth hypothesis (H5) of this study shows that the *t* = 7.133 indicates a positive relationship between perceived behavioral control and willingness to treat viral-infected patients through emotion-focused coping.

The sixth hypothesis (H6) demonstrated that the insignificant relationship between attitudes and willingness to treat viral-infected patients mediates by emotion-focused coping, where the (*t* = 0.434).

The seventh hypothesis (H7) statistically shows that the (*t* = 4.055) confirmed that emotion-focused coping mediates the relationship between subjective norms and willingness to treat viral-infested patients.

Overall, perceived behavioral control and subjective norms positively affect the willingness to treat viral-infected patients through emotion-focused coping. In contrast, attitudes do not significantly affect the willingness to treat viral-infected patients through emotion-focused coping. Therefore, the overall mediating hypotheses results are presented in Table 6.

**Table 6:** Mediating hypotheses.

	Original sample (O)	*T*-value	*P*-values
Perceived behavioral control → Emotion-focused coping → Willingness to treat viral infected patients	0.354	7.133	0
Attitudes → Emotion-focused coping → Willingness to treat viral infected patients	0.02	0.434	0.664
Subjective norms → Emotion-focused coping → Willingness to treat viral infected patients	0.303	4.055	0

## 5 Discussion

The present study aims to investigate and conclude the direct and indirect relationship between perceived behavioral control, attitudes, subjective norms, emotion-focused coping, and willingness to treat viral-infected patients among healthcare social workers. Overall findings show mixed results on the factors influencing healthcare workers’ readiness and decision-making in challenging healthcare contexts.

First, perceived behavioral control is a critical determinant of healthcare workers’ willingness to treat viral-infected patients (Shaikh et al., 2022). The significant positive relationship indicates that healthcare social workers who feel more confident and in control of their emotional ability to manage patient care are more inclined to express readiness to treat viral infections. This finding highlighted the importance of self-efficacy in healthcare settings, particularly during infectious disease outbreaks where effective patient management is essential. In this regard, Shubayr et al. (2020) suggested that interventions to enhance healthcare workers’ perceived control over their emotional actions and decisions could bolster their preparedness and resilience in responding to public health emergencies.

Second, in contrast to perceived behavioral control, attitudes toward treating viral-infected patients did not significantly predict emotion-focused coping to treat (Aschwanden et al., 2021). This unexpected result suggests that while healthcare workers may hold personal beliefs and evaluations about patient care, these attitudes may not directly translate into their willingness to treat infected patients (Alshehri et al., 2024). The lack of a significant relationship between attitudes and emotion-focused coping strategies suggests that healthcare workers’ evaluations of treating patients are not strongly tied to how they manage emotional responses. This could be attributed to the nature of coping, which may be influenced more by individual psychological traits, situational stress, or institutional support than evaluative attitudes. Healthcare workers might rely on pre-established coping mechanisms irrespective of their views on treating patients, especially in high-stress environments like pandemics.

Theoretically, this study highlights the importance of distinguishing between fear-based and positive attitude components in measuring attitudes. Emotional biases, heightened by stress, may skew responses, reflecting fear rather than genuine evaluative intentions. Practically, healthcare institutions must address these emotional barriers through psychological support, training, and resource adequacy to realign attitudes with the willingness to treat. Future research should integrate multidimensional attitude scales and consider contextual stressors to provide a nuanced understanding of these relationships.

This finding of the present study challenges conventional assumptions about the importance of attitudes in shaping healthcare emotions. It highlights the need to explore the interplay between attitudes and other influencing factors, such as perceived control and subjective norms.

Third, subjective norms, reflecting perceived social pressures and expectations, significantly predict healthcare workers’ emotion-focused coping to treat viral-infected patients (Shaikh et al., 2022). This finding highlights the role of the social environment in shaping healthcare professionals’ behavioral intentions. Healthcare workers are influenced by the norms and expectations within their professional and social circles, which can encourage or discourage their willingness to engage in patient care during infectious disease outbreaks (Wu et al., 2020; Sims et al., 2022). Understanding and leveraging these social dynamics are crucial for fostering a supportive and encouraging environment that promotes healthcare workers’ emotional commitment to patient care amidst public health challenges.

Fourth, emotion-focused coping plays a significant role in influencing healthcare professionals’ willingness to treat viral-infected patients. Such a coping strategy involves managing the emotional distress of challenging situations rather than directly addressing the problem. In healthcare, especially when dealing with highly contagious and potentially dangerous viral infections, emotion-focused coping can help professionals navigate the emotional and psychological hurdles of treating such patients (Nikolaev et al., 2023). According to Riaz et al. (2020), emotion-focused coping allows healthcare professionals to regulate their emotional responses, reducing anxiety, fear, and stress associated with treating viral-infected patients. By managing these emotions, professionals can maintain a calmer, more composed state crucial for effective patient care. Regular engagement in emotion-focused coping strategies, such as seeking social support, engaging in relaxation techniques, or practicing mindfulness, can enhance mental resilience (Nikolaev et al., 2023).

Fifth, a strong positive relationship between perceived behavioral control and willingness to treat viral-infected patients through the mediator of emotion-focused coping. Findings suggest that when healthcare professionals feel confident in their ability to manage and treat viral infections, they are more likely to use emotion-focused coping strategies, which enhances their willingness to treat such patients. This significance emphasizes empowering healthcare professionals with skills, training, and resources to boost their perceived control over their actions (Riaz et al., 2020).

Sixth, the findings of this study suggest that positive attitudes toward treating viral-infected patients do not necessarily lead to an increased willingness to treat them through emotion-focused coping. It might imply that attitudes alone, without the support of coping mechanisms or other factors, are insufficient to influence healthcare professionals’ willingness to engage in challenging clinical tasks (Limbu et al., 2020; Sims et al., 2022).

Seventh, a substantial positive relationship between subjective norms and willingness to treat viral-infected patients through emotion-focused coping. Overall findings suggest that when healthcare professionals perceive intense social pressures or support to treat viral-infected patients, they are more likely to engage in emotion-focused coping, which increases their willingness to treat such patients (Olum et al., 2020; Hernández-Fernández and Meneses-Falcón, 2022). This highlights the role of social influence and the importance of creating a supportive and encouraging work environment.

## 6 Implications

### 6.1 Implications for practice and policy

These findings of the present study have important implications for healthcare practice and policy. Firstly, interventions to strengthen healthcare workers’ perceived behavioral control, such as training programs focused on infection control measures and patient management strategies, could enhance their confidence and preparedness in handling viral-infected patients. Secondly, efforts to cultivate supportive and normative environments within healthcare settings may encourage greater willingness among healthcare workers to participate in patient care during infectious disease outbreaks. Strategies could include promoting team cohesion, providing adequate resources and support, and addressing safety and risk management concerns.

### 6.2 Implications for policy development

Policymakers can use the study’s findings to inform evidence-based policies to enhance healthcare workforce resilience and preparedness during infectious disease outbreaks. Policies should prioritize investments in healthcare infrastructure, including adequate supply chains for personal protective equipment (PPE), robust infection prevention and control measures, and comprehensive healthcare worker training programs. By implementing policies that support healthcare workers’ perceived behavioral control and address influential subjective norms, governments and healthcare organizations can strengthen the healthcare system’s capacity to respond effectively to public health emergencies.

Furthermore, policy initiatives should aim to promote interdisciplinary collaboration and knowledge exchange across healthcare sectors. Engaging stakeholders from public health, epidemiology, behavioral sciences, and healthcare administration can facilitate a holistic approach to pandemic preparedness and response. By fostering partnerships and leveraging expertise from diverse disciplines, policymakers can develop innovative strategies and adaptive solutions that enhance healthcare workforce readiness and mitigate the impact of infectious disease outbreaks on healthcare delivery systems.

## 7 Conclusion

This study aimed to determine if medical professionals were willing to treat COVID-19 patients during the pandemic. Therefore, to thoroughly test all the potential indicators that might motivate healthcare professionals to treat COVID-19 patients during the pandemic, the structural model using the theory of planned behavior was expanded in this paper by incorporating five constructs (i.e., perceived behavioral control, attitudes, subjective norms, emotion-focused coping, and willingness to treat viral infected patients). A survey was conducted in Riyadh, Saudi Arabia, using PLS-SEM approaches to test the assumptions. Overall results indicate that healthcare staff’s willingness to treat COVID-19 patients is favorably impacted by perceived behavioral control, subjective norms, and emotion-focused coping, where attitudes show negative results. Conversely, regarding the impression of the pandemic’s efficacy and the perceived danger of successfully shaping the willingness of healthcare workers to treat COVID-19 patients in Saudi Arabia, the Ministry of Health and the Government of Saudi Arabia should pay more attention to these indicators. It should also be emphasized how important it is to restructure and set up healthcare professionals’ training programs so they can learn how to minimize the perceived danger and effectiveness of the pandemic. As a result, these actions may eventually encourage physicians to treat patients during a national pandemic.

## 8 Limitations and future directions

While this study provides valuable insights, several limitations should be considered. The findings are based on a specific sample and context, which may limit their generalizability to other healthcare settings or regions. Future research could explore these relationships across diverse populations and healthcare disciplines to validate the findings and identify potential cultural or contextual variations. Additionally, longitudinal studies could investigate how these factors evolve over time and in response to different phases of infectious disease outbreaks, providing a deeper understanding of healthcare workers’ adaptive behaviors and decision-making processes.

## Data Availability

The original contributions presented in the study are included in the article/Supplementary material, further inquiries can be directed to the corresponding author.

## References

[ref1] Abdel Wahed W. Y. Hefzy E. M. Ahmed M. I. Hamed N. S. (2020). Assessment of knowledge, attitudes, and perception of health care workers regarding COVID-19, a cross-sectional study from Egypt. J. Community Health 45, 1242–1251. doi: 10.1007/s10900-020-00882-0, PMID: 32638199 PMC7340762

[ref2] Ajzen I. (1991). The theory of planned behavior. Organ. Behav. Hum. Decis. Process. 50, 179–211. doi: 10.1016/0749-5978(91)90020-T

[ref3] Aleanizy F. S. Alqahtani F. Y. Alanazi M. S. Mohamed R. A. Alrfaei B. M. Alshehri M. M.. (2021). Clinical characteristics and risk factors of patients with severe COVID-19 in Riyadh, Saudi Arabia: a retrospective study. J. Infect. Public Health 14, 1133–1138. doi: 10.1016/j.jiph.2021.07.014, PMID: 34343963 PMC8317445

[ref4] Alharbi A. A. Alqassim A. Y. Gosadi I. M. Aqeeli A. A. Muaddi M. A. Makeen A. M.. (2021). Regional differences in COVID-19 ICU admission rates in the Kingdom of Saudi Arabia: a simulation of the new model of care under vision 2030. J. Infect. Public Health 14, 717–723. doi: 10.1016/j.jiph.2021.04.012, PMID: 34020211 PMC8113109

[ref5] AlMazeedi S. M. AlHasan A. J. M. S. AlSherif O. M. Hachach-Haram N. Al-Youha S. A. Al-Sabah S. K. (2020). Employing augmented reality telesurgery for COVID-19 positive surgical patients. J. Br. Surgery 107, e386–e387. doi: 10.1002/bjs.11827, PMID: 32700761 PMC7404839

[ref6] Alshehri A. M. Alqahtani W. H. Moaili A. A. Almogbel Y. S. Almalki Z. S. Alahmari A. K.. (2024). An analysis of the intention of female pharmacy students to work in community pharmacy settings in Saudi Arabia using the theory of planned behavior. Saudi Pharm. J. 32:101996. doi: 10.1016/j.jsps.2024.101996, PMID: 38414782 PMC10897891

[ref7] Aschwanden D. Strickhouser J. E. Sesker A. A. Lee J. H. Luchetti M. Stephan Y.. (2021). Psychological and behavioural responses to coronavirus disease 2019: the role of personality. Eur. J. Personal. 35, 51–66. doi: 10.1002/per.2281, PMID: 32836766 PMC7361622

[ref9] Conner M. Sparks P. (2005). Theory of planned behaviour and health behaviour. Predicting Health Behav. 2, 121–162.

[ref9004] Crockett S. A. (2012). A five-step guide to conducting SEM analysis in counseling research. Counseling Outcome Research and Evaluation, 3, 30–47.

[ref10] Fishbein M. Ajzen I. (2010). Predicting and changing behavior: The reasoned action approach. London: Psychology Press.

[ref9002] Godbersen H. Hofmann L. A. Ruiz-Fernández S. (2020). How people evaluate anti-corona measures for their social spheres: attitude, subjective norm, and perceived behavioral control. Front. psychol, 11, 567405. 33281669 10.3389/fpsyg.2020.567405 PMC7689201

[ref11] Hair J. F. Jr. Matthews L. M. Matthews R. L. Sarstedt M. (2017). PLS-SEM or CB-SEM: updated guidelines on which method to use. Int. J. Multivar. Data Analysis 1, 107–123. doi: 10.1504/IJMDA.2017.087624

[ref12] Hernández-Fernández C. Meneses-Falcón C. (2022). “The worst thing that has happened to me”: healthcare and social services professionals confronting death during the COVID-19 crisis. Front. Public Health 10:957173. doi: 10.3389/fpubh.2022.957173, PMID: 35968471 PMC9374276

[ref13] Huynh G. Han N. T. N. Ngan V. K. Van Tam V. Le An P. (2020). Knowledge and attitude toward COVID-19 among healthcare workers at district 2 hospital, Ho Chi Minh City. Asian Pac J Trop Med 13, 260–265. doi: 10.4103/1995-7645.280396

[ref9003] Irfan M. Elavarasan R. M. Hao Y. Feng M. Sailan D. (2021). An assessment of consumers willingness to utilize solar energy in China: End-users’ perspective. Journal of Cleaner Production, 292, 126008.

[ref14] Johns R. (2010). Likert items and scales. Survey Question Bank Methods Fact Sheet 1, 11–28.

[ref15] Kaye A. D. Okeagu C. N. Pham A. D. Silva R. A. Hurley J. J. Arron B. L.. (2021). Economic impact of COVID-19 pandemic on healthcare facilities and systems: international perspectives. Best Pract. Res. Clin. Anaesthesiol. 35, 293–306. doi: 10.1016/j.bpa.2020.11.009, PMID: 34511220 PMC7670225

[ref16] Khan M. A. Khan M. I. Illiyan A. Khojah M. (2021). The economic and psychological impacts of COVID-19 pandemic on Indian migrant workers in the Kingdom of Saudi Arabia. Healthcare 9:1152. doi: 10.3390/healthcare9091152 34574926 PMC8464826

[ref17] Ko N. Y. Feng M. C. Chiu D. Y. Wu M. H. Feng J. Y. Pan S. M. (2004). Applying theory of planned behavior to predict nurses' intention and volunteering to care for SARS patients in southern Taiwan. Kaohsiung J. Med. Sci. 20, 389–398. doi: 10.1016/S1607-551X(09)70175-5, PMID: 15473650 PMC7129400

[ref18] Limbu D. K. Piryani R. M. Sunny A. K. (2020). Healthcare workers’ knowledge, attitude and practices during the COVID-19 pandemic response in a tertiary care hospital of Nepal. PLoS One 15:e0242126. doi: 10.1371/journal.pone.0242126, PMID: 33156873 PMC7647104

[ref19] Matta D. Herring P. Beeson W. L. Wiafe S. (2023). The role of perceived susceptibility, perceived severity, perceived barriers and benefits in COVID-19 vaccine hesitancy and uptake among outpatient surgery nurses in the United States: a qualitative study. Int. J. Transl. Med. Res. Pub. Health 7, 1–8. doi: 10.21106/ijtmrph.439

[ref20] McCready J. L. Nichol B. Steen M. Unsworth J. Comparcini D. Tomietto M. (2023). Understanding the barriers and facilitators of vaccine hesitancy towards the COVID-19 vaccine in healthcare workers and healthcare students worldwide: An umbrella review. PLoS One 18:e0280439. doi: 10.1371/journal.pone.0280439, PMID: 37043505 PMC10096263

[ref21] Minuye B. Alebachew W. Kebede M. Asnakew S. Mesfin Belay D. (2021). Intention to care for COVID-19 patients among nurses working at health care institutions of Debre Tabor town, north Central Ethiopia. Risk Manage. Healthcare Policy 14, 2475–2481. doi: 10.2147/RMHP.S311830, PMID: 34163266 PMC8214202

[ref22] Montano D. E. Kasprzyk D. (2015). Theory of reasoned action, theory of planned behavior, and the integrated behavioral model. Health Behav. Theor. Res. Prac. 70:231.

[ref23] Ness M. M. Saylor J. Di Fusco L. A. Evans K. (2021). Healthcare providers' challenges during the coronavirus disease (COVID-19) pandemic: a qualitative approach. Nurs. Health Sci. 23, 389–397. doi: 10.1111/nhs.12820, PMID: 33580590 PMC8012981

[ref9001] Nguyen T. M. Le G. N. H. (2021). The influence of COVID-19 stress on psychological well-being among Vietnamese adults: The role of self-compassion and gratitude. Traumatology, 27, 86.

[ref24] Ngwewondo A. Nkengazong L. Ambe L. A. Ebogo J. T. Mba F. M. Goni H. O.. (2020). Knowledge, attitudes, practices of/towards COVID 19 preventive measures and symptoms: a cross-sectional study during the exponential rise of the outbreak in Cameroon. PLoS Negl. Trop. Dis. 14:e0008700. doi: 10.1371/journal.pntd.0008700, PMID: 32886678 PMC7497983

[ref25] Nikolaev B. N. Lerman M. P. Boudreaux C. J. Mueller B. A. (2023). Self-employment and eudaimonic well-being: the mediating role of problem-and emotion-focused coping. Entrep. Theory Pract. 47, 2121–2154. doi: 10.1177/10422587221126486

[ref26] Olum R. Chekwech G. Wekha G. Nassozi D. R. Bongomin F. (2020). Coronavirus disease-2019: knowledge, attitude, and practices of health care workers at Makerere University teaching hospitals, Uganda. Front. Public Health 8:181. doi: 10.3389/fpubh.2020.00181, PMID: 32426320 PMC7204940

[ref27] Patwary M. M. Bardhan M. Disha A. S. Hasan M. Haque M. Z. Sultana R.. (2021). Determinants of COVID-19 vaccine acceptance among the adult population of Bangladesh using the health belief model and the theory of planned behavior model. Vaccine 9:1393. doi: 10.3390/vaccines9121393, PMID: 34960138 PMC8707510

[ref28] Riaz S. Saleem Y. Hazrat H. Ahmed F. Sajid U. Qadri S. F.. (2020). Mental health outcomes and coping strategies among health care workers exposed to coronavirus disease 2019 (COVID-19). Int. J. Endorsing Health Sci. Res. 8, 56–66. doi: 10.29052/JEHSR.v8.i2.2020.56-66

[ref29] Saleem M. Kamarudin S. Shoaib H. M. Nasar A. (2022). Retail consumers’ behavioral intention to use augmented reality mobile apps in Pakistan. J. Internet Commer. 21, 497–525. doi: 10.1080/15332861.2021.1975427

[ref30] Saleem M. Kamarudin S. Shoaib H. M. Nasar A. (2023). Influence of augmented reality app on intention towards e-learning amidst COVID-19 pandemic. Interact. Learn. Environ. 31, 3083–3097. doi: 10.1080/10494820.2021.1919147

[ref31] Saqlain M. Munir M. M. Rehman S. U. Gulzar A. Naz S. Ahmed Z.. (2020). Knowledge, attitude, practice and perceived barriers among healthcare workers regarding COVID-19: a cross-sectional survey from Pakistan. J. Hosp. Infect. 105, 419–423. doi: 10.1016/j.jhin.2020.05.007, PMID: 32437822 PMC7211584

[ref32] Shaikh D. Kamarudin S. Rizal A. M. Shoaib H. M. (2022). Willingness of healthcare workers to treat COVID-19 patients during the pandemic: extended theory of planned behavior. Probl. Perspect. Manag. 20, 210–223. doi: 10.21511/ppm.20(4).2022.16

[ref33] Sharma K. Joshi A. Poudyal S. Khatiwada K. Dhakal S. Neupane H. C. (2020). Emotions and coping strategies of health care workers working in different hospitals of Chitwan during COVID-19 pandemic. J. Chitwan Med. College 10, 9–15. doi: 10.54530/jcmc.272

[ref34] Shoaib H. M. Saleem M. (2023). “An online market in your pocket: how does an augmented reality application influence consumer purchase decision” in Technological sustainability and business competitive advantage. eds. Mubarak M. Hamdan A. (Cham: Springer International Publishing), 307–313.

[ref35] Shubayr M. A. Mashyakhy M. Al Agili D. E. Albar N. Quadri M. F. (2020). Factors associated with infection-control behavior of dental health–care workers during the covid-19 pandemic: a cross-sectional study applying the theory of planned behavior. J. Multidiscip. Healthc. 13, 1527–1535. doi: 10.2147/JMDH.S278078, PMID: 33209032 PMC7669526

[ref36] Sims H. Alvarez C. Grant K. Walczak J. Cooper L. A. Ibe C. A. (2022). Frontline healthcare workers experiences and challenges with in-person and remote work during the COVID-19 pandemic: a qualitative study. Front. Public Health 10:983414. doi: 10.3389/fpubh.2022.983414, PMID: 36203659 PMC9531651

[ref37] Sin C. S. Rochelle T. L. (2022). Using the theory of planned behaviour to explain hand hygiene among nurses in Hong Kong during COVID-19. J. Hosp. Infect. 123, 119–125. doi: 10.1016/j.jhin.2022.01.018, PMID: 35124145 PMC8812086

[ref38] Snoubar Y. Zengin O. (2022). Fear of being infected with COVID-19 virus among the medical social workers and its relationship to their future orientation. Front. Psychol. 13:985202. doi: 10.3389/fpsyg.2022.985202, PMID: 36148097 PMC9485880

[ref39] Spoorthy M. S. Pratapa S. K. Mahant S. (2020). Mental health problems faced by healthcare workers due to the COVID-19 pandemic–a review. Asian J. Psychiatr. 51:102119. doi: 10.1016/j.ajp.2020.102119, PMID: 32339895 PMC7175897

[ref40] Tripathi R. Alqahtani S. S. Albarraq A. A. Meraya A. M. Tripathi P. Banji D.. (2020). Awareness and preparedness of COVID-19 outbreak among healthcare workers and other residents of south-West Saudi Arabia: a cross-sectional survey. Front. Public Health 8:482. doi: 10.3389/fpubh.2020.00482, PMID: 33014977 PMC7461899

[ref41] Wong K. K. K. (2013). Partial least squares structural equation modeling (PLS-SEM) techniques using SmartPLS. Mark. Bull. 24, 1–32.

[ref42] Wu A. W. Buckle P. Haut E. R. Bellandi T. Koizumi S. Mair A.. (2020). Supporting the emotional well-being of health care workers during the COVID-19 pandemic. J. Patient Safety Risk Manage. 25, 93–96. doi: 10.1177/2516043520931971

[ref44] Zhong E. H. Smiley R. O’Hara C. Martin B. (2024). Healthcare on the go: a comparative analysis profiling the travel nurse workforce in the United States. J. Nurs. Regul. 15, 88–97. doi: 10.1016/S2155-8256(24)00032-2

