# Supplementary material for: Understanding the willingness of healthcare workers to treat viral infected patients in Saudi Arabia: evidence from post-COVID-19 pandemic
Source: Front Sociol. 2025 Mar 4;10:1461479. doi: 10.3389/fsoc.2025.1461479 (PMC11913814; doi:10.3389/fsoc.2025.1461479)
Supplement: Supplementary file 1 [file Table_1.docx]

Supplementary Material

## Supplementary Table

**Appendix A Meaurment Items and Operational definitions**

| **Constructs** | **Operational Definition** |
| --- | --- |
| Perceived Behavioral Control | In the context of the COVID-19 pandemic, perceived behavior control refers to the individuals’ perception of COVID-19 patients. In this study, perceived behavior control has defined the medical social workers' perception and controllability to consider easiness and/or difficulty while treating COVID-19 patients. |
|  |  |
|  |  |
|  |  |
| Attitudes | In the context of the COVID-19 pandemic, attitudes refer to the personal valuation of an individual that holds a positive or negative response towards COVID-19 patients. In this research study, attitudes have defined the medical social workers' response and valuation toward treating COVID-19 patients. |
|  |  |
|  |  |
|  |  |
|  |  |
| Subjective Norms | In the context of the COVID-19 pandemic, the subjective norms refer to the perceived social (family and friends) pressure faced by an individual towards COVID-19 patients that reference whether people would agree to be in contact with COVID-19 patients or not. In this research study, the subjective norm has defined the medical social workers' decision to treat COVID-19 patients with the consent of a social group. |
|  |  |
|  |  |
|  |  |
| Emotion-Focused Coping | Emotion-focused coping involves regulating individuals' positive or negative feelings and emotional responses to the problem instead of addressing the problem. In the present study, emotion-focused coping defines the medical social workers' emotional response toward treating COVID-19 patients. |
